# Data augmentation-based conditional Wasserstein generative adversarial network-gradient penalty for XSS attack detection system

**DOI:** 10.7717/peerj-cs.328

**Published:** 2020-12-14

**Authors:** Fawaz Mahiuob Mohammed Mokbal, Dan Wang, Xiaoxi Wang, Lihua Fu

**Affiliations:** 1 College of Computer Science, Faculty of Information Technology, Beijing University of Technology, Beijing, China; 2 Faculty of Computer Science, ILMA University, Karachi, Pakistan; 3 State Grid Management College, Beijing, China

**Keywords:** Data augmentation, Conditional-Wasserstein generative adversarial net, Imbalance dataset, XSS Attack, Web applications security

## Abstract

The rapid growth of the worldwide web and accompanied opportunities of web applications in various aspects of life have attracted the attention of organizations, governments, and individuals. Consequently, web applications have increasingly become the target of cyberattacks. Notably, cross-site scripting (XSS) attacks on web applications are increasing and have become the critical focus of information security experts’ reports. Machine learning (ML) technique has significantly advanced and shown impressive results in the area of cybersecurity. However, XSS training datasets are often limited and significantly unbalanced, which does not meet well-developed ML algorithms’ requirements and potentially limits the detection system efficiency. Furthermore, XSS attacks have multiple payload vectors that execute in different ways, resulting in many real threats passing through the detection system undetected. In this study, we propose a conditional Wasserstein generative adversarial network with a gradient penalty to enhance the XSS detection system in a low-resource data environment. The proposed method integrates a conditional generative adversarial network and Wasserstein generative adversarial network with a gradient penalty to obtain necessary data from directivity, which improves the strength of the security system over unbalance data. The proposed method generates synthetic samples of minority class that have identical distribution as real XSS attack scenarios. The augmented data were used to train a new boosting model and subsequently evaluated the model using a real test dataset. Experiments on two unbalanced XSS attack datasets demonstrate that the proposed model generates valid and reliable samples. Furthermore, the samples were indistinguishable from real XSS data and significantly enhanced the detection of XSS attacks compared with state-of-the-art methods.

## Introduction

Over the last decade, the worldwide web has grown exponentially, and web applications are increasingly being deployed to provide sustainable and accessible services to the public. These have attracted the attention of governments, companies, and individuals. Similarly, cyberattacks on web applications are increasing; consequently, increasing the severity of web applications and users’ risks. Cross-site scripting (XSS) attack is one of the prevalent and growing attacks on web applications. Successful XSS attacks lead to various degrees of consequences for users, governments, and businesses. For the user, XSS attacks can be used to steal sensitive user information such as user credentials and session tokens or impersonate the user to carry out authorized actions on behalf of the user. For businesses and governments, XSS attacks can be used to change the appearance or behavior of target websites and steal confidential information. These authorities may face dire consequences, including loss of reputation, legal battles, and financial losses (Deepa & Thilagam, 2016). Cybercriminals exploit security vulnerabilities within web applications often caused by several factors, including the level of application programmers’ experience in security and inheriting vulnerabilities from open-source and third-party packages. These security vulnerabilities could allow cybercriminals to inject malicious content into the HTML trusted pages displayed to end-users (Sarmah, Bhattacharyya & Kalita, 2018).

State-of-the-art XSS attack detection systems are applied on the server-side, client-side, or both. The analysis methods used to distinguish between malignant and benign payload could be static, dynamic, or hybrid (Sarmah, Bhattacharyya & Kalita, 2018). However, these methods have limitations, such as low detection rate (DR), high false positive (FP)/negative rates, and often not scalable over time (Mitropoulos & Spinellis, 2017). Therefore, they are inefficient, especially with emerging techniques and evolving forms of XSS payloads developed continuously by cybercriminals (Lekies et al., 2017; Zhou & Wang, 2019).

In 2019, XSS attacks became the most widespread attack vectors. Approximately 40% of cyberattacks have been attributed to XSS attacks, according to Precise Security research (Precise Security, 2020), which is expected to increase in the future significantly. Furthermore, the overall number of new XSS vulnerabilities in 2019 (2,023) increased by 79.20% compared with that in 2017 (1,129) as per the National Vulnerabilities Database (National Institute of Standards and Technology, 2020). Additionally, there are various reports and warnings from information security experts in Industrial Control Systems Vulnerabilities Statistics (Andreeva et al., 2016). Many studies in related literature used FP as metrics to measure model accuracy instead of DR, which reveals the effect of unbalanced data and can be expensive in the cybersecurity domain (Elkan, 2001). Technically, the DR represents the effective detection of attacks and is a critical factor in detection systems. When the DR is not clearly defined, it raises concerns about the cybersecurity system’s effectiveness. Consequently, there is an increase in the number of major risks unidentified by various tools/models (Deepa & Thilagam, 2016; Lekies et al., 2017).

Existing machine learning (ML) techniques are proven to be highly efficient in handling security challenges. The algorithms are trained using data of previously known behaviors (supervised learning), and each class of behavior is recognized to be either anomalous or legitimate. However, web pages are a mixture of multiple languages such as JavaScript and HTML, which were formally unstandardized, enabling the use of various coding techniques that are susceptible to attacks. Therefore, XSS attacks have peculiar and irregular characteristics; further, the volume of labeled data on XSS attacks with up-to-date cases is limited and highly unbalanced (Obimbo, Ali & Mohamed, 2017; Nunan et al., 2012; Mokbal et al., 2019).

Consequently, applying most standard ML algorithms to XSS data in the straightforward are unsuitable and challenging compared with other well-developed clean data domains (Vluymans, 2019; Mokbal et al., 2019). To our best knowledge, the limited and unbalanced data of XSS cyber-attack based on ML learning have not been addressed in the literature, which is worth to study. The detection system is invariably affected by the class imbalances problem. Specifically, ML algorithms focus on maximizing accuracy, which technically means that all misclassified errors are handled equally and uniformly, implying that the algorithms do not handle unbalanced datasets even if they are accurate and clean (Vluymans, 2019). A learning algorithm may discard instances of the minority class in the dataset in such a problem. The attack samples are often of the minority class and handled as noise while recognizing only samples of the majority class (Buda, Maki & Mazurowsk, 2018). Therefore, the ML-based model design should consider the dataset’s weight and evaluation criteria (Vluymans, 2019).

The traditional methods for addressing the challenges of limited and unbalanced data that could be used are oversampling the minority class or undersampling the majority class. Yet, each method has its limitations. Oversampling can lead to overfitting, whereas undersampling may discard useful data, which subsequently leads to loss of information (Vluymans, 2019).

To mitigate the challenges of limited and highly unbalanced XSS attack dataset, we proposed a data augmentation method based on the conditional GAN and Wasserstein GAN with a gradient penalty (C-WGAN-GP). Our proposed method aims to achieve the minority class’s oversampling using a more robust generative approach to rebalancing the training dataset by adding identical and valid samples to the minority class. Samples generated based on the minority class’s overall distribution are generalized using the C-WGAN-GP generative network instead of local information as the traditional methods do.

The generative adversarial network (GAN) (Goodfellow et al., 2014) is considered a potential solution to the challenges described above. It is a type of a deep generative model that aims to learn the joint probability distribution of samples and labels from training data, which can be further used for several applications such as predictor training, classifier, and data augmentations (Pan et al., 2019).

The main contributions of this study can be summarized as the following:

• We proposed the WGAN-based adversarial training with conditional minority class (attack labels) to generate valid and indistinguishable samples of real XSS attack data. To preserve various features covering the data space range and enable the generator to learn the original data space distribution, we pass the upper and lower data space to the conditional generator. Furthermore, the augmented data are not added to the real training data arbitrarily; the process is performed only if the generated sample $\tilde {x}$ satisfies the critic. Thus, it ensures that the added samples are identical to the real data and improving the training data.
• We further propose a boosting classification model using the XGBoost algorithm trained on the augmented training dataset generated by C-WGAN-GP, which significantly improved the attack detection efficiency.
• The proposed method is evaluated with two real and large unbalance XSS attack datasets. Experiments show that our proposed augmentation framework generates valid samples indistinguishable from real XSS data and outperformed state-of-the-art methods with XSS attacks detection. Although we presented the proposed framework formally for XSS attack detection, it can be generalized and extended to other applications areas.

The rest of this study is presented as follows: In ‘Related Work’, we gave the most related literature. In ‘Proposal and experimental methodology’, we introduced the model design and the methodology of experiments. We presented the results and discussion in ‘Results and Discussion’. ‘Conclusions’ is the conclusion and future work.

## Related Work

Web applications have become part of our everyday lives and have achieved significant success and substantial financial gains for organizations; consequently, ML-based XSS attacks detection has gained much attention from the research community. However, there are challenges in using ML-based methods, including finding or designing an adequate, accurate, and balanced dataset designed for ML algorithms usage. Unfortunately, there is no public and standard dataset intended for this purpose (Obimbo, Ali & Mohamed, 2017; Nunan et al., 2012; Mokbal et al., 2019), where researchers can create their datasets based on their requirements and orientation.

The authors (Rathore, Sharma & Park, 2017) proposed a classifier model against XSS attacks for social sites using their dataset, which consists of 100 samples collected from XSSed, Alexa, and Elgg. They applied multiple algorithms and achieved the best results by using the random forest algorithm. However, the dataset used to train the algorithm is small, possibly selective, and may not reflect the real attacks. Moreover, the DR score of 0.949 was considered inadequate.

Wang et al. (2017) proposed a hybrid analysis method to detect malicious web pages by extracting and using three sets of features: URL, HTML, and JavaScript. The reported DR was 88.20%, implying that the method fails to detect 11.80% of real threats.

Another research work (Wang, Cai & Wei, 2016) proposed a deep learning model (stacked denoising autoencoder) to detect malicious codes. They used sparse random projections to reduce the dimensions of the data. Despite the model’s complexity, the DR score was 0.9480, which is inadequate for detecting malicious attacks. Moreover, the model has a high FP rate of 4.20% with a high computational cost.

Wang et al. (2014) used an ensemble learning method (ADTree and AdaBoost) to detect XSS attacks. However, the DR score of 0.941 is inadequate, with a high FP rate of 4.20%. Mokbal et al. (2019) proposed a scheme based on dynamic feature extraction and deep neural network to detect XSS attacks. Using their developed dataset, they achieved an estimated DR of 98.35%. However, the model is a deep neural network, which has potential high computational costs.

Multiple studies (López et al., 2013; Haixiang et al., 2017) have thoroughly investigated the problem of unbalanced data. The problem can be mitigated at two levels: first, at the model level, by modifying existing algorithms to focus more on the minority class, such as embedding cost-sensitive methods with an ensemble learning algorithm, and second, at the data level, by preprocessing data before it is fed into the algorithm (López et al., 2013). The data-level approach uses either the undersampling or the oversampling method. Undersampling mitigates class imbalance by randomly removing some samples from the majority class in the training dataset. Conversely, oversampling mitigates the class imbalance by duplicating some minority class samples in the training dataset. However, these methods can result in the loss of important information and overfitting, respectively. Kovács et al. (2002) proposed the synthetic minority oversampling technique (SMOTE) as an oversampling method for the minority class. However, this method generates an equal number of synthetic samples for each real sample from the minority class without considering neighbor samples.

Consequently, the overlap between class increases with the potential generation of noisy samples (Vluymans, 2019). Adaptive versions of SMOTE have been proposed, including borderline SMOTE and DBSMOTE. Borderline SMOTE (Han, Wang & Mao, 2002) concentrates synthetic samples along the borderline within classes. Cluster-based algorithm DBSMOTE (Bunkhumpornpat, Sinapiromsaran & Lursinsap, 2012) assembles data samples into clusters using DBSCAN clustering and adaptive synthetic sampling (He et al., 2008). However, these methods are based on local information instead of on the overall-minority class distribution (Douzas & Bacao, 2018).

## Proposal and Experimental Methodology

This section presents the different generative networks, including GAN, CGAN, WGAN, and WGAN-GP, in addition to our proposed model. The model architecture, experimental methodology design, XGBoost attack detector, and datasets are also presented as follows.

### GANs

GAN is recently introduced as a novel approach to train a generative model, which has achieved success in different fields, including images and natural language processing (Goodfellow et al., 2014). The network comprises two adversarial models: first, the generative model *G* for learning the distribution of data and, second, the discriminator *D*, which estimates the probability that a sample is from the real training data instead of *G*. Both models *G* and *D* in the network compete to outsmart the other where *G* and *D* can be a nonlinear mapping function, such as a multilayer perceptron. The generator *G* learns the distribution *p*_*g*_ over data *x* and constructs a mapping function from noise space of uniform dimension *p*_*z*_(*z*) to data space as *G*(*z*, *θ*_*g*_). The discriminator *D*(*x*, *θ*_*d*_) returns a single scalar to estimate the probability that an instance *x* came from the real data distribution rather than *p*_*g*_.

Both *G* and *D* are trained together, such that the parameters for G are adjusting to minimize *log*(1 − *D*(*G*(*z*))) and parameters for *D* are adjusting to minimize *logD*(*X*), similarly to the two-player minimax game accompanied by value function *V*(*G*, *D*) as in Eq. (1).

(1) \begin{eqnarray*}\min _{G}\max _{D}V \left( G,D \right) ={\mathrm{E}}_{x\sim {P}_{r}} \left[ logD \left( x \right) \right] +{\mathrm{E}}_{x^{\sim }\sim {P}_{g}} \left[ log \left( 1-D \left( G \left( z \right) \right) \right) \right] \end{eqnarray*}

A CGAN extends GAN by adding space *y* to both the *G* and *D* to control data generation. The additional space *y* could be supplied from real data (class label) or data from other sources (Mirza & Osindero, 2014). The training phase of both CGAN and GAN is similar, and the minimax objective function of *D* and *G* is as shown in Eq. (2).

(2) \begin{eqnarray*}\min _{G}\max _{D}V \left( G,D \right) ={\mathrm{E}}_{x\sim {P}_{r}} \left[ logD \left( x{|}y \right) \right] +{\mathrm{E}}_{x^{\sim }\sim {P}_{g}} \left[ log \left( 1-D \left( G \left( z{|}y \right) ,y \right) \right) \right] \end{eqnarray*}

where *p*_*r*_ is the real data distribution and *p*_*g*_ is the CGAN model distribution implicitly defined as $\tilde {x}= \left( z,y \right) ,z\sim p(z),y\sim p(y)$, where y and noise z are combined as input to the hidden layer.

The CGAN and GAN use Jensen–Shannon (JS) divergence shown in Eq. (3) to measure generative samples.

(3) \begin{eqnarray*}JS \left( {p}_{r},{p}_{g} \right) =KL({p}_{r}{|}{|}{p}_{m})+KL({p}_{g}{|}{|}{p}_{m}), \left\{ \begin{array}{@{}l@{}} \displaystyle kl~is~ullback-Leibler~divergence\\ \displaystyle {p}_{m}=({p}_{r}+{p}_{g})/2 \end{array} \right. \end{eqnarray*}

However, both GAN and CGAN have unstable training (vanishing gradients) and mode collapse problems (Pan et al., 2019). To overcome these problems, the WGAN optimizes the original GAN objective using Wasserstein-1 distance, also known as the earth-mover distance (EMD) instead of *JS* (Arjovsky, Chintala & Bottou, 2017), where EMD measures the distance between the actual distribution of the data and the distribution of the generative model as in Eq. (4).

(4) \begin{eqnarray*}W \left( {p}_{r},{p}_{g} \right) =\begin{array}{@{}l@{}} \displaystyle inf\\ \displaystyle \gamma \in \prod ({p}_{r},{p}_{g}) \end{array}{E}_{ \left( x,y \right) \sim \gamma } \left[ \left\vert \left\vert x-y \right\vert \right\vert \right] \end{eqnarray*}

where ∏(*p*_*r*_, *p*_*g*_) represents all possible joint distribution sets (*x*, *y*) of *p*_*r*_ and *p*_*g*_ of real and generated data distribution, respectively. Such that, for each feasible joint distribution *γ*, a real instance *x* and a generated instance *y* can be sampled, and the instance distance [||*x* − *y*||] is calculated. Therefore, the expected value *γ* ${E}_{ \left( x,y \right) \sim \gamma }[{|}{|}x-y{|}{|}]$ of the instance to the distance under the joint distribution *γ* can be calculated.

The value function of WGAN was obtained by utilizing Kantorovich–Rubinstein duality (Villani, 2009), as shown in Eq. (5).

(5) \begin{eqnarray*}\min _{G}\max _{D\in \mathcal{F}}V \left( G,D \right) =\mathrm{E}_{x\sim {p}_{r}} \left[ D \left( x \right) \right] -\mathrm{E}_{x^{\sim }\sim {p}_{g}} \left[ D \left( x^{\sim } \right) \right] \end{eqnarray*}

where $\mathcal{F}$ is the set of 1-Lipschitz functions restricted by k, $ \left\vert \mathcal{F} \left( \mathrm{x} \right) -\mathcal{F} \left( \mathrm{y} \right) \leq \mathrm{k} \right\vert \mathrm{x}-\mathrm{y}{|}$, and *p*_*g*_i is the model distribution. The value function is minimized concerning G determined by $\tilde {x}=G(z),z\sim p(z)$. Therefore, the discriminator called a critic minimizes the *W*(*p*_*r*_, *p*_*g*_). Nevertheless, the WGAN still faces gradient extinction or gradient explosion because of weight clipping in the discriminator. The gradient penalty (*GP*) was added to the total loss function in the WGAN distance discriminator to achieve training stability (Gulrajani et al., 2017). The new objective value function is adjusted, as shown in Eq. (6).

(6) \begin{eqnarray*}\min _{G}\max _{D}V \left( G,D \right) =\mathrm{E}_{x\sim {p}_{r}} \left[ D \left( x \right) \right] -\mathrm{E}_{x^{\sim }\sim {p}_{g}} \left[ D \left( x^{\sim } \right) \right] -\lambda {E}_{\hat {x}\sim {p}_{\hat {x}}} \left[ ({\nabla }_{\hat {x}}D \left( \hat {x} \right) {|}{{|}}_{2}-1)^{2} \right] \end{eqnarray*}

where $\hat {x}=\varepsilon x+ \left( 1-\varepsilon \right) ,\tilde {x}$ is a convex function combination of real data distribution *p*_*r*(*x*)_ and the model data distribution *p*_*g*(*z*)_, $\varepsilon \sim unform \left[ 0,1 \right] $, whereas *λ* is the gradient penalty parameter.

### C-WGAN-GP model

This study proposes using data augmentation based on a GAN that takes real samples as inputs and outputs adversarial samples. The learning algorithm of our proposed model is based on CGAN (Mirza & Osindero, 2014) and WGAN-GP (Gulrajani et al., 2017), where both networks are integrated. Precisely, we used the WGAN-GP optimization approach to optimize CGAN. The integrated generative network is called C-WGAN-GP. Our goal is to generate synthetic samples of attack class (minority) with identical distribution to real XSS attack scenarios.

The primary idea is to use the learning of the joint probability distribution over *x* samples and *y* labels from the training data to perform data augmentation only if the generated sample $\tilde {x}$ satisfy the critic. The problem of unbalanced data can be mitigated by using an augmented data in the classification tasks, therefore improving the robustness and performance of the XSS attack detection for unbalanced data. A well-trained generator with joint distributions set (*x*, *y*) of *p*_*r*_ and *p*_*g*_ of real and generated data distribution optimized using GP should be able to generate $(\tilde {x},y)$ samples within the tolerated latent space and identical to the original data of (*x*, *y*), therefore providing valuable information to the detector as additional training data. To ensure that only useful instances are added to augment the training dataset, only generated cases that satisfy the critic are added to the original data.

The *y* labels of the minority class, which are XSS attacks in our case, are used as a conditional parameter. Passing the upper and lower real data space to the generator provides the generator with additional auxiliary information to define the latent space. The latent space establishes the scope of samples in the data variance. Therefore, the generator using the auxiliary latent space generates samples within the tolerated latent space identical to the real data. Consequently, the discriminator distinguishes the synthetic samples as real within small feedback loops needed to train the generator, reducing computational cost while providing high-quality generated data.

In the discriminator *D*, the *p*_*r*_ and *p*_*g*_ are linked with *y* in a joint hidden layer representation, whereas in generator *G*, the *y* is combined with *p*(*z*) in the same manner. The objective minimax function of models *D* and *G* is as shown in Eq. (7), whereas Eqs. (8) and (9) represent the loss reduction functions of *D* and *G*, respectively.

(7) \begin{eqnarray*}\min _{G}\max _{D\in \mathcal{F}}V \left( G,D \right) =\mathrm{E}_{x\sim {p}_{r}} \left[ D(x{|}y) \right] -\mathrm{E}_{x^{\sim }\sim {p}_{g}} \left[ D \left( x^{\sim }{|}y \right) \right] -\lambda {E}_{\hat {x}\sim {p}_{\hat {x}}} \left[ ({\nabla }_{\hat {x}}D \left( \hat {x}{|}y \right) {|}{{|}}_{2}-1)^{2} \right] \end{eqnarray*}

(8) \begin{eqnarray*}\mathrm{min~ }L \left( D \right) =\mathrm{E}_{x^{\sim }\sim {p}_{g}} \left[ D \left( x^{\sim }{|}y \right) \right] -\mathrm{E}_{x\sim {p}_{r}} \left[ D \left( x{|}y \right) \right] +\lambda .{E}_{\hat {x}\sim {p}_{\hat {x}}} \left[ ({\nabla }_{\hat {x}}D \left( \hat {x}{|}y \right) {|}{{|}}_{2}-1)^{2} \right] \end{eqnarray*}

(9) \begin{eqnarray*}\mathrm{min~ }L \left( G \right) =-\mathrm{E}_{x^{\sim }\sim {p}_{g}} \left[ D \left( x^{\sim }{|}y \right) \right] \end{eqnarray*}

### Generative model design

Generative networks have gained popularity in image data; however, we are interested in digital datasets. Therefore, the technique used is similar but differs in design and implementation. In our model, we did not apply convolutional layers.

In the generator model *G*, the concatenate input layer equal to (z + c), where z is the vectors of noise set within the range of batch size and data dimension. The c is the conditioning variable’s dimension that equals 1. The model has three hidden layers of the neural network—the number of units in hidden layers equal to 128, 256, and 512, respectively. The output layer equal to z and the concatenate layer is equal to the input layer (z + c).

In the discriminator (critic) *D*, the same architecture is used but in descending order of hidden layers, which equal to 512, 256, 128, respectively. The third layer is a linear activation function that equals to 1. The batch size and numbers of epochs for the network are 128 and 4000, respectively. The activation function used for the generator and discriminator is the rectified linear unit. The C-WGAN-GP is fitted using Adam optimizer with α, β1, and β2 parameters calibrated to le-4, 0.5, and 0.99, respectively. The alpha or (α) for short refers to the learning rate, while beta1 (β1) and beta2 (β2) refer to the exponential decay rate for the first-moment and second-moment estimates, respectively. The value of the *GP* coefficient *λ* for C-WGAN-GP is set to 10. The parameter *k* of *D* is tuned to 4, whereas *k* of *G* is tuned to 1. A conditional critic neural network is trained to approximate the EMD using the minority class for control mode up to 4000 training steps. Note that the parameters are defined empirically, whereas the performance reduces significantly by changing the parameters.

The other different hyperparameters not mentioned are consistent with those originally reported. During the testing phase, the generated samples added to the real dataset are the samples approved by the critic. Algorithm 1 presents the generative approach for XSS attack data. Note that the other generator network architectures are similar, with negligible differences, which may be necessary for the implementation.

BOX 1 The C-WGAN-GP algorithm.

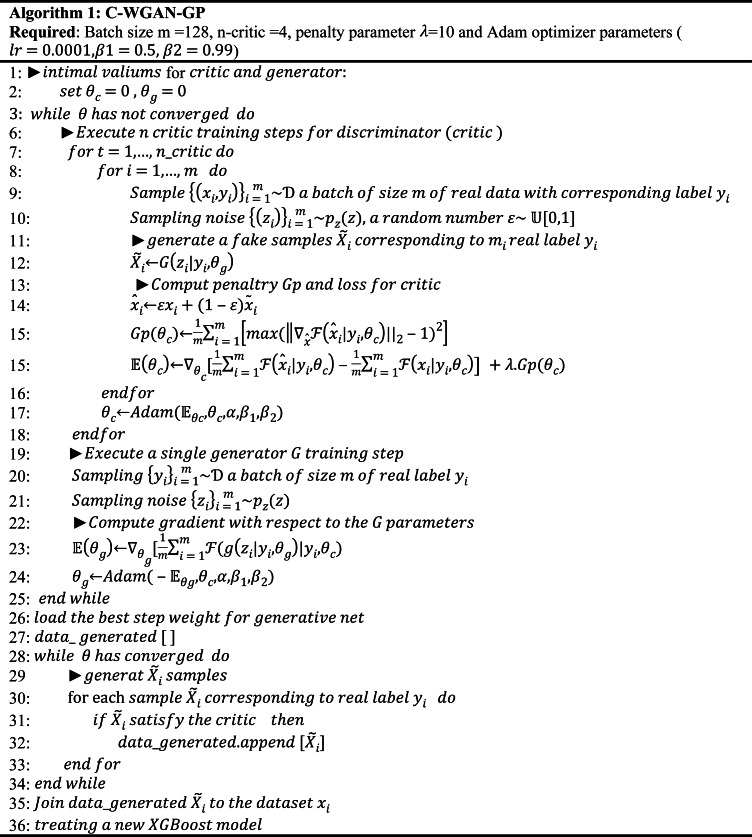

### Experimental methodology

This study proposed generative model C-WGAN-GP, an oversampling solution of minority class to solve unbalanced XSS attack data. We trained the detector (XGBoost) using the real training dataset and test it using the test dataset before without augmented data, and then the results were recorded for comparison. Subsequently, we trained each of the GANs models using real training dataset to generate synthetic data. We repeated the training of the detector using the augmented data and test it using the test dataset. The results of each model were recorded for comparison. Similarly, traditional oversampling methods were trained and tested.

**Figure 1:**
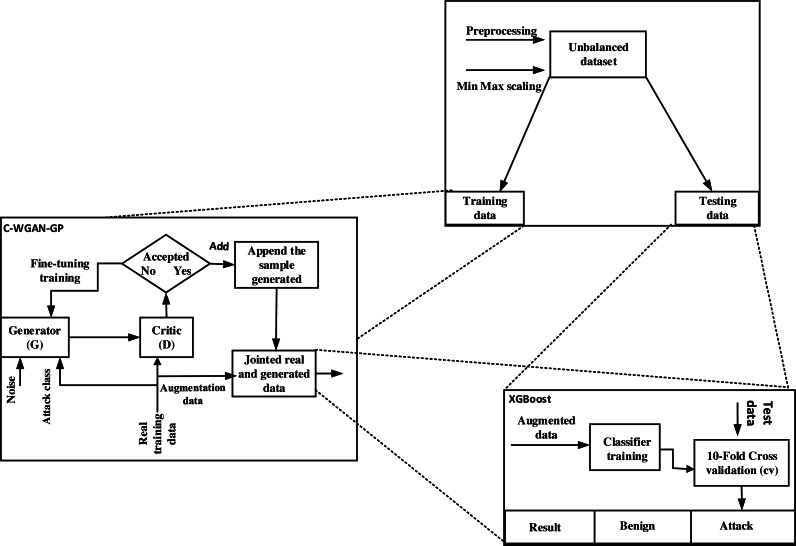
Systemic flowchart of C-WGAN-GP.

The C-WGAN-GP generator performance was evaluated in two directions. First, we assessed the performance of the C-WGAN-GP generative adversarial network against the other four GANs. Second, we compared our C-WGAN-GP model with two traditional oversampling methods include SMOTE (Han, Wang & Mao, 2002) and adaptive synthetic (ADASYN) (Bunkhumpornpat, Sinapiromsaran & Lursinsap, 2012). The systemic flowchart of the proposal is shown in Fig. 1.

### Detector

We use an external model to evaluate the quality of the data generated by our proposed method and other methods. The XGBoost boosting model was used for all experiments to assess the augmented data’s quality on XSS attack detection performance. The XGBoost is a state-of-the-art boosting algorithm that is simple to apply and interpret, is highly efficient, does not require advanced data preparation, and has many advanced functions (Chen & Guestrin, 2016). The algorithm’s learning rate, tree size, and tree number hyperparameters tuned to 0.3, 4, and 100, respectively.

### Datasets

To our knowledge, there is only one public dataset for intrusion detection that includes XSS attacks that we were able to find called CICIDS2017 designed by the Canadian Institute of Cybersecurity (Sharafaldin, HabibiLashkari & Ghorbani, 2018). The released CICIDS2017 dataset contains 80 features that include regular traffic and recent common attacks. To provide attack features, we used a CICFlowMeter tool to extract features from PCAPs files and used Selenium with Damn Vulnerable Web Application to run automatic XSS attacks. However, there are only 652 XSS attacks traffic, and over 140,008 regular traffic, making it a highly unbalanced dataset.

We have added another dataset proposed in our previous work (Mokbal et al., 2019). The dataset includes 67 features with labels that are categorized based on three groups, including HTML, JavaScript, and URL. We extracted 1,000 XSS attack samples and 100,000 benign samples as a second dataset.

We applied data preprocessing to each dataset. In both datasets, all samples have two classes, XSS attack and benign, which are set to [1, 0], respectively. The two datasets were split randomly into training and test sets with a 70% and 30% ratio, respectively, where data augmentation was performed only on the training dataset. Missing and infinite values in CICIDS2017 were updated using their features’ mean values, whereas the zero features and duplicate rows were omitted. The number of features with clean data in the CICIDS2017 dataset is 78, and the number of features with clean data in the second dataset is 67. Subsequently, the data’s scale within the range [0, 1] was applied using the minimax function for both datasets. The class-level distribution of datasets is shown in Table 1. The datasets are available at https://doi.org/10.6084/m9.figshare.13046138.v4.

**Table 1:** The class-level distribution of the datasets.

ID	Dataset	#Samples		#Attributes		Minority class		Majority class		#Minority samples		#Majority samples	
1	CICIDS2017 (Sharafaldin, HabibiLashkari & Ghorbani, 2018)	140,660	77		Attack		Benign		652		140,008		
2	MLPXSS (Mokbal et al., 2019)	101,000	67		Attack		Benign		1000		100,000		

### Performance evaluation criteria

Although many performance metrics have been introduced, GANs do not have an objective measure of the generator model, as there is no consensus on the best metric that captures the strength/limitations of the model and should be used for a fair comparison between models (Borji, 2019). We used *precision*, $detectio{n}_{Rate} \left( DR \right) $/ recall, and *F*1 − *score*, which are proven and widely adopted methods for quantitatively estimating the quality of discriminative models suggested by Google Brain research (Lucic et al., 2018). Precision measures the generated instances similarity to the real ones on average. Whenever the instances generated are similar to the real instances, the precision is high. In GANs, the recall (detection rate) measures diversity. A high recall indicates the generator can generate any instances found in the training dataset. For cybersecurity detection sys, the recall/detection rate denotes the ability of sys to detect the real attacks. The F-score reflects the harmonic mean of precision and recall. Further, the area under the curve (AUC) measure that demonstrates a detector’s ability to distinguish between classes and summarizes the detector’s performance is also collected. The measurements are defined as follows.

(10) \begin{eqnarray*}Precision= \frac{TP}{ \left( TP+FP \right) } \end{eqnarray*}

(11) \begin{eqnarray*}DR= \frac{TP}{ \left( TP+FN \right) } \end{eqnarray*}

(12) \begin{eqnarray*}F-Score=2 \left( \frac{ \left( Recall\times Precision \right) }{ \left( Recall+Precision \right) } \right) \end{eqnarray*}

(13) \begin{eqnarray*}AUC= \frac{1}{2} \left( \frac{TP}{TP+FN} + \frac{TN}{TN+FP} \right) \end{eqnarray*}

## Results and Discussion

The C-WGAN-GP-based data augmentation approach was implemented in Python 3.7 using the TensorFlow framework on Linux operating system. The proposed method was implemented alongside four other GAN-based generative methods and two traditional generative methods (SMOTE and ADASYN). All the methods were validated using 10-fold cross-validation. To demonstrate how the attack DR decreased as the gap between normal and malicious classes increased, we injected different ratios of the majority class in the training data to train the XGBoost detector model using AUC and DR criteria. The model tested on the fixed test dataset size of 30% each time. During the attack detection test, the results show that DR decreased from 96.59% with an injected ratio of 2% of the majority class to approximately 91.00% with an injected ratio of 100% of the majority class. These results are shown in Table 2.

Using our generative approach, we injected the generated data into a real training dataset to create a new augmented training dataset. The augmented data were used to train the XSS attack detector, which is different from the generative framework to judge the data quality. The detector was tested using the real test dataset. This experiment mechanism was applied to all of the methods we used, and results were reported for each scenario.

The average results reported in each trial on the CICIDS2017 dataset are shown in Table 3 (Sharafaldin, HabibiLashkari & Ghorbani, 2018). Using the same mechanism, we repeated the experiments using the dataset extracted from our previous work (Mokbal et al., 2019), and the results are shown in Table 4.

Notably, using augmented data generated by the proposed method significantly improved the XSS attacks detection compared with state-of-the-art baseline methods. The results in Tables 3 and 4 show that the DR increased up to 98.95% in the CICIDS2017 dataset and up to 96.67% in the second dataset. That is, our generative model able to generate any sample found in the XSS attack training dataset. The precision measure is also high, which equals 0.99333 on the first dataset and 0.989761 on the second dataset. The precision results imply that the proposed generative model generated samples look similar to the real XSS attack samples on average. Concerning F-Score, the proposed generative model was superior to other generative methods. It achieved the result score of 0.990382 in the first data set and the score of 0.978078 in the second dataset. The AUC measure of the proposed generative model is also continued to be outperformed the other methods in both datasets with a significant margin.

**Figure 2:**
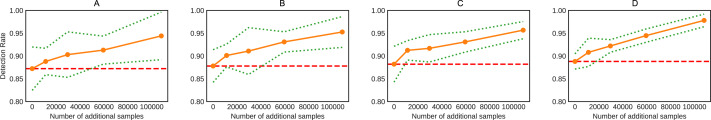
Effects of the data augmentation on the XSS attack detection rate over the baseline for GAN, WGAN, WGAN-GP, and C-WGAN-GP. (A) CGAN detection rate, (B) WGAN detection rate, (C) WGAN-GP detection rate, and (D) C-WGAN-GP detection rate. The red horizontal dashed line indicates the baseline estimate of each generative model.

The results for ADASYN, WGAN-GP, WGAN, SMOTE, and CGAN showed improved DR performance, respectively, with varying proportions. The effects of the additional samples on the XSS attack DR under the condition of acceptance by the discriminator on the CICIDS2017 dataset are shown in Fig. 2 along with standard deviation. The standard deviation of the C-WGAN-GP is overall small compared to other methods; it also decreases as training steps increase. While the standard deviation of CGAN and WGAN is not smooth and shows more variation. The standard deviation of WGAN-GP was smoother than GAN and WGAN but less smooth and more varied than C-WGAN-GP. This fact indicates the stability of C-WGAN-GP training to some extent. The results suggest that the C-WGAN-GP significantly outperformed CGAN, WGAN and WGAN-GP.

**Table 2:** The collapse of the detection rate as the unbalanced classes’ gap increased.

Ratio	Train-auc mean	Train-auc std	Train-recall mean	Train-recall std	Test-auc mean	Test-auc std	Test-recall mean	Test-recall std
2%	0.998624	0.000345	0.976264	0.004189	0.995596	0.004673	0.965895	0.008869
5%	0.997947	0.001006	0.966985	0.003804	0.994683	0.001888	0.954185	0.007851
14%	0.991259	0.004218	0.946233	0.003984	0.985622	0.009439	0.937748	0.023061
40%	0.966395	0.002146	0.927482	0.003458	0.965025	0.010199	0.922232	0.022333
100%	0.965471	0.001782	0.923503	0.005247	0.965422	0.007108	0.910094	0.011817

**Table 3:** Detection results using data augmented generated through different methods on the CICIDS2017 dataset.

Criteria	None	ADASYN	SMOTE	GAN	CGAN	WGAN	WGAN-GP	C-WGAN-GP
DR (sensitivity)	0.883333	0.98667	0.96000	0.92667	0.96333	0.97333	0.97833	0.987452
Specificity	0.929967	0.99983	0.99980	0.99993	0.99993	0.99987	0.99996	0.99993
Precision	0.90513	0.98339	0.97959	0.99286	0.99313	0.98649	0.98949	0.99333
F-score	0.894099	0.98502	0.96970	0.95862	0.97800	0.97987	0.983878	0.990382
AUC	0.917548	0.99325	0.97990	0.9633	0.98163	0.9866	0.989148	0.993691

**Table 4:** Detection results using data augmented generated through different methods on the second dataset.

Criteria	None	ADASYN	SMOTE	GAN	CGAN	WGAN	WGAN-GP	C-WGAN-GP
DR (sensitivity)	0.873333	0.956667	0.943343	0.932983	0.933333	0.94667	0.951211	0.966667
Specificity	0.979967	0.999833	0.999933	0.989867	0.999867	0.99973	0.999933	0.9999
Precision	0.956198	0.982877	0.992982	0.985915	0.985915	0.97260	0.977864	0.989761
F-score	0.912889	0.969595	0.967527	0.958719	0.958904	0.95945	0.964353	0.978078
AUC	0.92665	0.97825	0.970138	0.961425	0.9666	0.9732	0.975572	0.983283

The superiority of the C-WGAN-GP over the rest of the GANs is due to the fact that the model is enhanced with the characteristics of two generative networks, CGAN and WGAN-GP. The C-WGAN-GP used minority class labels that act as an extension to the latent space *z* to generate and discriminate instances well, which inspired from CGAN. Consequently, the model can learn a multi-modes mapping from inputs to outputs by feeding it with different contextual auxiliary information. The C-WGAN-GP optimized using Wasserstein distance with gradient penalty inspired by WGAN-GP. The training process is more stable and less sensitive to model design and configurations of hyperparameter. Further, the loss of the critic is related to the quality of instances created by the generator.

Precisely, the lower the critic’s loss when evaluating the instances generated, the higher the expected quality of the instances generated. This criterion is crucial because unlike other GANs that seek stability by finding a balance between two models, WGAN seeks convergence and minimizes generator loss. Furthermore, adding the generated samples of CGAN, WGAN, and WGAN-GP that satisfied the discriminator’s acceptance condition (critic) adds value to the augmented training dataset, which increases detector ability and efficiency.

**Figure 3:**
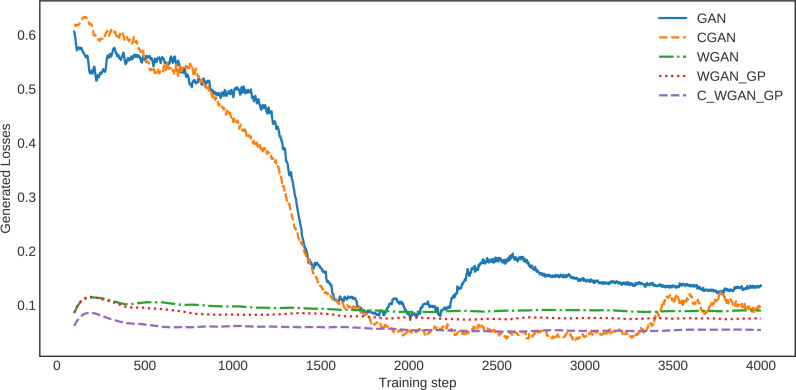
The loss of generated data.

The loss of generated data for C-WGAN-GP compared with that of the other four GAN methods is shown in Fig. 3. It is quite clear that the loss curve of C-WGAN-GP decreased regularly and continuously compared to all other generative methods. The loss curves of GAN and CGAN are unstable, and the models went to collapse mode during the generating phase. The WGAN and WGAN–GP loss curves decreased regularly; however, it is high compared with C-WGAN-GP. Note that GAN and CGAN are using JS divergence, whereas WGAN and C-WAN-GP are using the Wasserstein distance or EMD.

**Figure 4:**
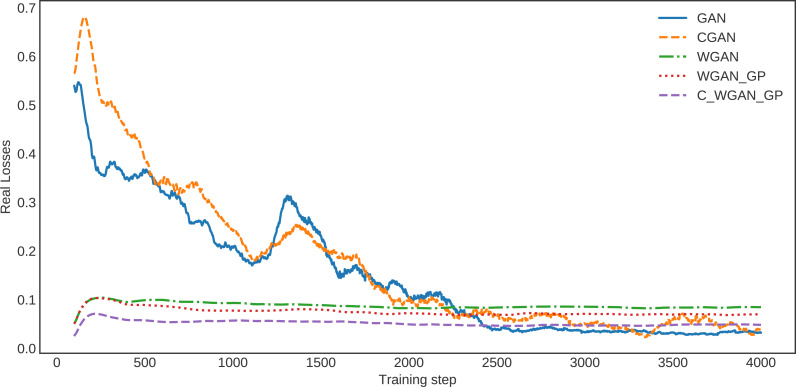
The loss of real data.

Similarly, in the loss curve of real data, the GAN and CGAN face difficulty learning the training data distribution. In contrast, the WGAN and WGAN–GP losses decreased regularly; however, it is high compared with C-WGAN-GP. The C-WGAN-GP seems to learn the training data distribution better than all other generative methods, as shown in Fig. 4.

**Figure 5:**
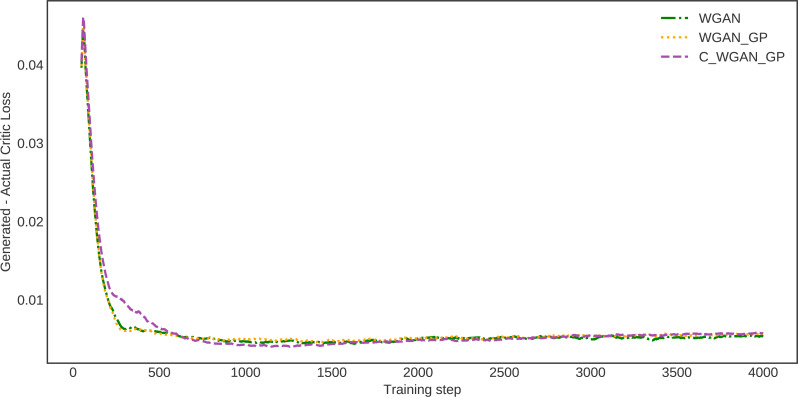
Difference between critic (EM distance estimate) loss on generated and real samples.

To estimate the proposed method’s generalization ability, we investigated the Wasserstein critic, in which the distance between actual and generated data losses is calculated. This estimate demonstrates how much the data generated by the proposed model and real data are identical. The difference in distance between the real and generated data distribution of WGAN, WGAN-GP, and C-WGAN-GP that generative models learn to minimize is shown in Fig. 5. The distance between generated and real data of C-WGAN-GP is close to zero. That is, The C-WGAN-GP generated samples that are identical to real data distribution; further, the training stability of the proposed generative model is adequate.

**Figure 6:**
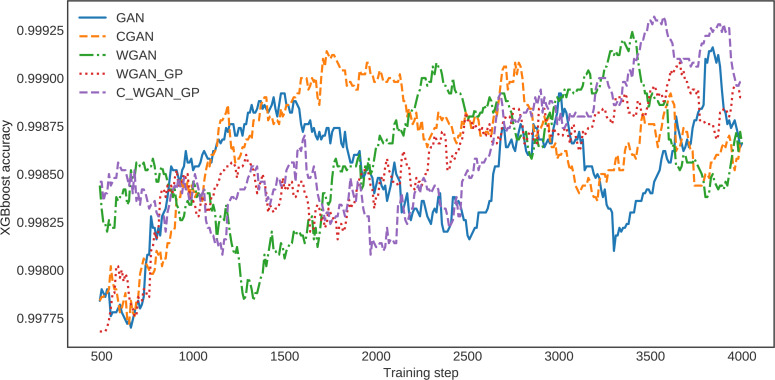
The detector loss function over various generated data.

**Figure 7:**
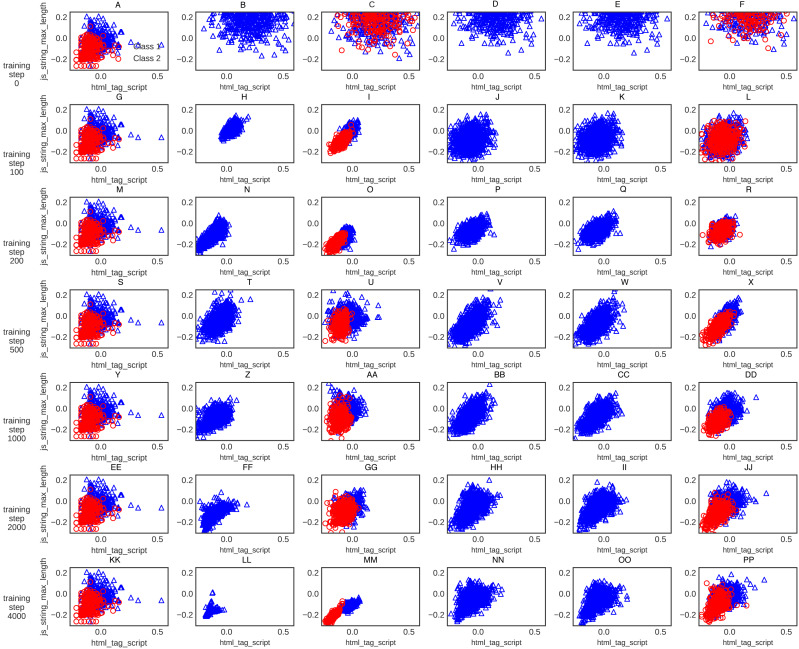
(A-PP) General visualization of sample generation for C-WGAN-GP compared to other generative methods.

For further clarification, the XGBoost classification accuracy trained on the five different generative methods’ data is shown in Fig. 6. The XGBoost accuracy curve of C-WGAN-GP data is higher than that of other models, which indicates the quality of the data generated by the proposed model. Figure 7 shows a general visualization example of the data quality generated by C-WGAN-GP compared with other generative methods and displays the collapse mode of GAN and CGAN between 2500 and 4000 of training steps in the second dataset. In addition to the beginning of the gradient extinction of WGAN at 4000 of training steps.

## Conclusions

This study proposed a conditional critic neural network with a gradient penalty called C-WGAN-GP to improve the XSS attack detection on unbalanced datasets. The C-WGAN-GP is trained to approximate the EM distance with an auxiliary of minority class for control mode to generate valid and reliable synthetic samples with identical distribution to real XSS attack scenarios. We trained a new boosting model using the augmented dataset to improve the XSS attack detection system and mitigate an unbalanced dataset problem. We conducted experiments to compare the proposed method with GAN, CGAN, WGAN, WGAN-GP, SMOTE, and ADASYN using two real-world XSS attack datasets. Experimental results show that the proposed method can train a generator model with improved training stability. The proposed method enhanced the detection of XSS attacks and prevented adversarial examples that have been widely used to target AI cyber defense systems. Furthermore, the C-WGAN-GP method can be extended to other forms of attacks and other fields, including the medical field, where datasets are highly unbalanced.

For future work, we will investigate network training stability to generate data using various designs over different network architectures. It is a significant problem worthy of further research.

##  Supplemental Information

10.7717/peerj-cs.328/supp-1 Supplemental Information 1 Raw datasets

10.7717/peerj-cs.328/supp-2 Supplemental Information 2 Preprocessing data in CSV format

10.7717/peerj-cs.328/supp-3 Supplemental Information 3 Preprocessing data (pickle format, Use Pickle from Python to open)

## Additional Information and Declarations

### Data Deposition

The following information was supplied regarding data availability: All raw data are available as Supplemental Files. Additional data are available at figshare: Mokbal, Fawaz (2020): Cross-Site Scripting Attack (XSS) dataset. figshare. Dataset. https://doi.org/10.6084/m9.figshare.13046138.v4.
